# Uncomplicated Extravascular Implantable Cardioverter‐Defibrillator Extraction After 4 Years Dwell Time: A Case Report

**DOI:** 10.1111/jce.16530

**Published:** 2024-12-18

**Authors:** Jolien A. de Veld, Kirsten M. Kooiman, Reinoud E. Knops

**Affiliations:** ^1^ Heart Center, Department of Clinical and Experimental Cardiology, Amsterdam Cardiovascular Sciences Amsterdam UMC location AMC, The Netherlands

**Keywords:** case report, extraction, extravascular ICD, implantable cardioverter‐defibrillator, lead

## Abstract

The extravascular implantable cardioverter‐defibrillator (EV‐ICD) was developed to overcome complications associated with transvenous leads while being able to deliver anti‐tachycardia pacing (ATP). The lead is implanted in the substernal space, which makes extraction a cautious procedure. We present a case of a 51‐year‐old women with a successful EV‐ICD extraction after a lead dwell time of 4 years, which is the longest reported. The EV‐ICD lead was extracted using simple traction after the removal of all adhesions at the xiphoidal site around the lead. We advise to only use extraction tools if the initial attempt is not successful and if no adhesions at the caudal part of the lead are visible anymore, as these tools could also damage the surrounding tissue and the lead.

## Graphical abstract

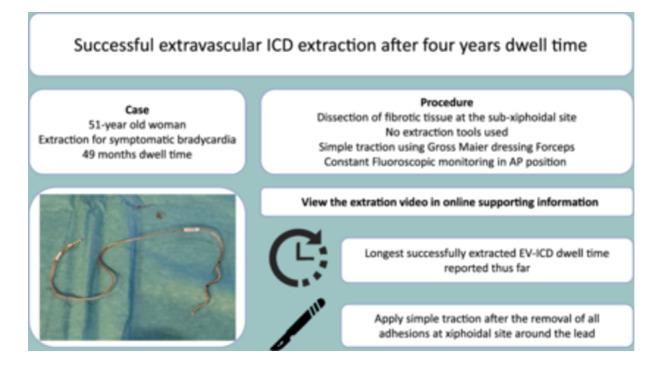

## 1 Introduction

Implantable cardioverter‐defibrillators (ICDs) are a safe and effective therapy for the prevention of sudden cardiac death [1]. Nevertheless, the traditional transvenous ICD (TV‐ICD) carries the risks for intravascular and intracardiac complications [2]. The subcutaneous ICD (S‐ICD) was developed to overcome these complications, but due to the extra‐thoracic design and a concomitant higher defibrillation threshold, the pulse generator of this device is twice the size of the TV‐ICD [3]. Additionally, the S‐ICD is not able to deliver anti‐tachycardia pacing (ATP). The extravascular ICD (EV‐ICD) was developed as an alternative to the TV‐ICD and S‐ICD by placing the lead in the substernal space, avoiding the vasculature [4]. Due to the close proximity to the heart, the EV‐ICD offers the benefit of ATP, and less energy is required to defibrillate the heart in comparison with the S‐ICD, making the generator size comparable to the TV‐ICD.

Due to the novelty of the EV‐ICD, data on lead extraction after long dwell times are scarce. A recent analysis reported safe lead removal up to 3 years after implantation but also mentioned one failed lead extraction after 58 months [5]. Besides this, extraction tools were not necessary in the majority of cases. To assess the risks associated with EV‐ICD lead extraction, data is needed for device and lead removal extending over this 3‐year experience.

## 2 Case Presentation

**Figure 1:**
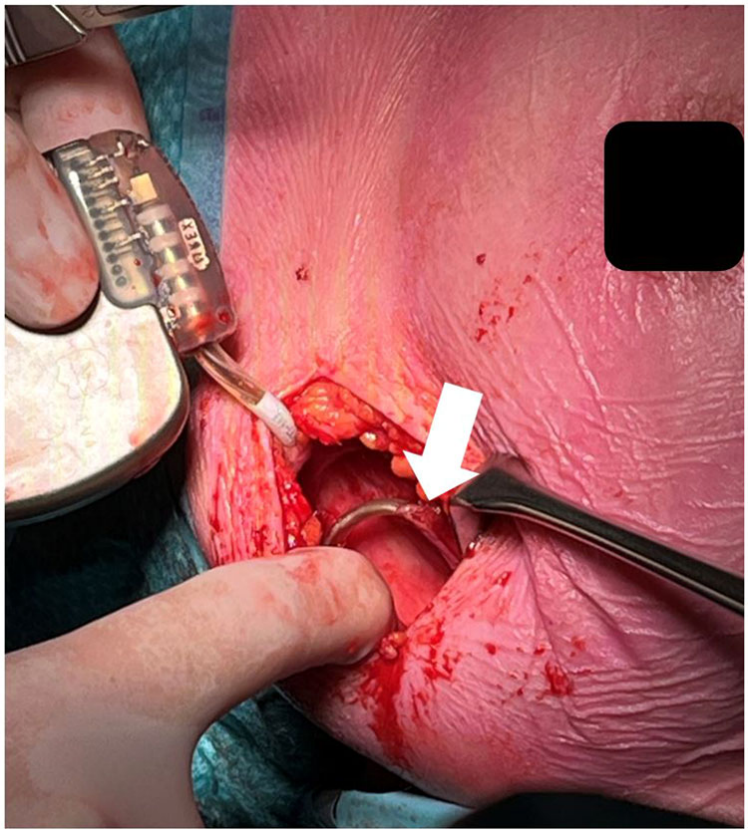
Illustrates the EV‐ICD generator pocket. The white arrow indicates the adhesion of the lead to the thoracic wall.

**Figure 2:**
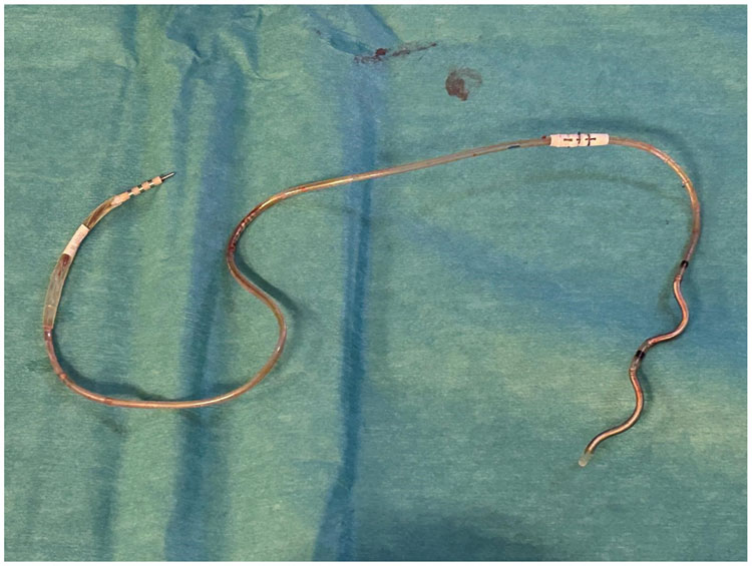
Illustrates the EV‐ICD lead after extraction. No adhesions are visible.

A 51‐year‐old woman received an EV‐ICD 4 years prior for primary prevention due to familial phospholamban mutation. She underwent EV‐ICD extraction and reimplantation with a dual chamber transvenous ICD due to a pacing indication for symptomatic bradycardias that developed during follow‐up after EV‐ICD implantation. Additionally, the patient had a history of pain located around the xiphoid radiating toward the pulse generator site since the device was implanted. At the time of extraction, the dwell time of the EV‐ICD was 49 months. During the procedure, the generator was easily removed, but we noticed that the lead showed significant adhesion to the thoracic wall (Figure 1). These adhesions were carefully dissected using blunt finger dissection and electrocautery. Subsequently, the xiphoid incision was opened and the proximal part of the lead was easily retracted from the pulse generator pocket to the Xiphoid incision. Next, the suture sleeve, which was secured to the fascia with three nonabsorbable sutures, was released by cutting the sutures with a surgical blade. Subsequently, sub‐xiphoidal fibrotic tissue surrounding the lead was carefully dissected with Mcindoe Scissors to release the part of the lead superior to the suture sleeve. Then, simple traction was applied to the lead with Gross Maier dressing Forceps to retract the lead from the sub‐sternal space (Figure 2). Simultaneous fluoroscopic monitoring in the anteroposterior (AP) position confirmed safe extraction with no bleeding or damage to the heart (Supporting Information Video). After closure of the xiphoid and midaxillary incisions, a transvenous dual chamber ICD was successfully implanted left‐sided. There were no complications after 16 days of follow‐up.

## 3 Discussion

To our knowledge, this case represents a successful EV‐ICD extraction with the longest dwell time to date. Previous literature, including data from Sagi et al., described successful lead removals with shorter dwell times but also noted one failed extraction after 58 months in which a 6.4 cm portion of the lead was abandoned in the substernal space [5]. In that case, lead removal was complicated by diaphragmatic adhesions, which made the need for extraction tools necessary. The mechanical and laser sheaths caused damage to the lead, which led to a broken lead. In the nine cases reported by Sagi et al. with a dwell time greater than 1 year, extraction tools were used in five cases, whereas only simple traction was utilized in cases with a lead dwell time of less than 1 year. This suggests that more extensive adhesions could be present after longer periods of time, which might make physicians more careful in the extraction process. Nevertheless, no notable adhesion formation within the substernal space was reported in our case and the cases described by Sagi et al. The adhesions were only being present at the xiphoid incision and in the pulse generator pocket. In our case, the adhesions located near the suture sleeve were dissected using a scalpel and Mcindoe Scissors, while also removing the non‐resorbable sutures from the sleeve. During the initial phase of lead extraction, slightly more force was required to extract the first part of the lead; however, this did not cause any damage to the heart or the substernal space. We therefore suggest to carefully remove all adhesions around the lead at the xiphoid incision site, whereafter simple traction should be performed on the lead by using, for example, a Gross Maier dressing forceps to maintain a constant grip. Extraction tools such as extraction or laser sheets should only be used if the initial attempt is not successful and if no adhesions at the caudal part of the lead are visible anymore, as these tools could also damage the surrounding tissue and fracture the lead.

### 3.1 Extravascular Compared to Transvenous and Subcutaneous Lead Removal

The procedure of EV‐ICD removal is comparable to the S‐ICD, with simple traction also being the standard procedure in the latter [6]. Besides this, both EV‐ICD and S‐ICD have a lumenless design, resulting in an extraction method that differs from a TV‐ICD, where the lumen in the lead is used to introduce an extraction stylet.

Nevertheless, the functionality of the EV‐ICD is more comparable to the TV‐ICD, as the device is able to deliver ATP. The extraction of transvenous leads is associated with serious complications, including mortality [7]. In the cases that are currently known of EV‐ICD extractions, no complications were observed, even though in some cases (part of) the lead was abandoned in the substernal space. The EV‐ICD is, therefore, a safe alternative for patients with an ICD indication without the need for bradycardia pacing to avoid complications associated with transvenous leads. What should be kept in mind, is that most complications in transvenous devices occur after longer periods of time and data of the EV‐ICD with a follow‐up period of > 3 years are not available yet [8, 9].

## 4 Conclusions

EV‐ICD extraction can be performed safely up to 4 years after implantation. This can be achieved by applying simple traction to the lead with forceps after removing all adhesions at the xiphoidal site around the lead. Further data on EV‐ICD extractions after longer lead dwell times are necessary.

## Author Contributions

All authors contributed to the study conception and design. Material preparation, data collection, and analysis were performed by Jolien A. de Veld, Kirsten M. Kooiman, and Reinoud E. Knops. The first draft of the manuscript was written by Jolien A. de Veld, and all authors commented on previous versions of the manuscript. All authors read and approved the final manuscript.

## Consent

The participant has consented to the submission of the case report and their photographs and videos to the journal. Written informed consent was obtained for publication of the data presented in this paper.

## Conflicts of Interest

K.M.K. reports consultancy fees from Boston Scientific. R.E.K reports consultancy fees and research grants from Abbott, Boston Scientific, Medtronic, and Cairdac and has stock options from AtaCor Medical Inc. The other author declares no conflicts of interest.

## Supporting information


**Supplementary Video EV‐ICD lead extraction**.
